# Ulnar Artery Reconstruction: Lateral Circumflex Femoral Arterial Graft in the Setting of Hypothenar Hammer Syndrome

**Published:** 2019-01-14

**Authors:** Nicholas C. Oleck, Stephen L. Viviano, Ashley Ignatiuk

**Affiliations:** Department of Surgery, Division of Plastic and Reconstructive Surgery, Rutgers New Jersey Medical School, Newark

**Keywords:** hypothenar hammer syndrome, arterial graft, ulnar hammer syndrome, microsurgery, hand surgery

## CASE DESCRIPTION

**Figure 1:**
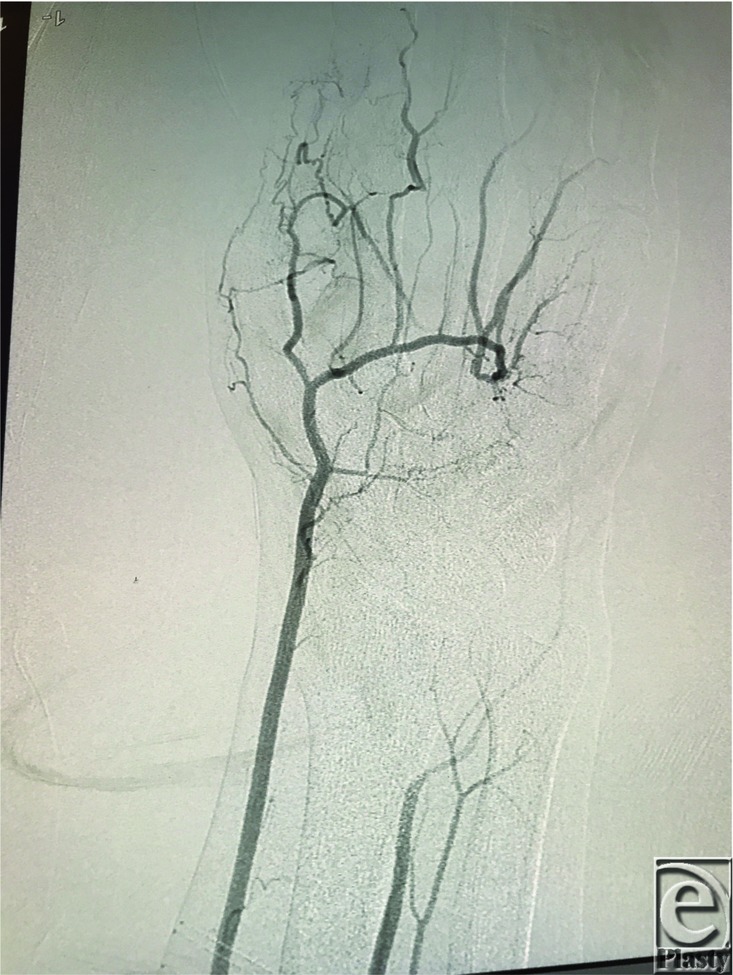
Preoperative angiogram of the right upper extremity showing complete occlusion of the ulnar artery.

A 35-year-old left-handed man who works as a mechanic and firefighter presented to our institution with hand pain and cold intolerance for 3 months. He was referred by a local vascular surgeon who had made the diagnosis of hypothenar hammer syndrome (HHS) following an angiogram of the right upper extremity showing complete occlusion of the ulnar artery (Fig 1). On initial examination, Doppler signals of the ulnar artery, superficial palmar arch, and ring/small digital arteries were absent in the right hand. Subsequently, the patient developed an ulcer on his right long fingertip after minor trauma that had not healed over a 4-week period. He suffered ischemic hand pain and severe cold intolerance. After conservative management failed, the decision was made for operative intervention. We proposed using an arterial graft versus a venous graft in order to improve long-term patency. Our plan was to utilize the descending branch of the lateral circumflex femoral artery (DLCFA) as an arterial graft.

Intraoperatively, Doppler ultrasound revealed a segmental thrombotic defect extending from the superficial palmar arch, across the Guyon canal, extending 10 cm proximally in the forearm. The ulnar artery was resected back to healthy bleeding pulsatile flow, resulting in a segmental defect of 12 cm. Final pathology of the submitted ulnar artery segment revealed organizing thromboembolus in the lumen, which at the time of resection was adherent to the lumen.

The DLCFA was harvested, with care taken to prevent injury to the lateral femoral cutaneous nerve. A 12-cm segment of the artery was circumferentially dissected, as well as approximately 2 cm of 2 branching vessels at the distal end of the segment that would be used to reconstruct the superficial palmar arch and common digital arteries with one graft. With the arterial graft completely dissected, it was ligated both distally and proximally.

**Figure 2:**
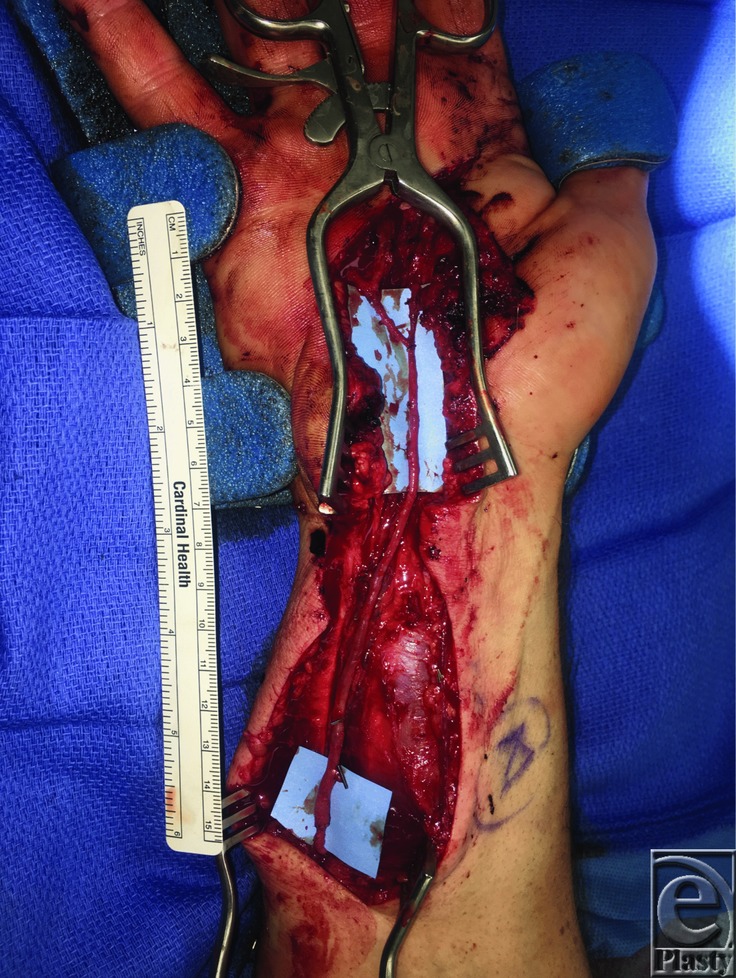
Lateral circumflex femoral arterial graft inset intraoperatively, measuring approximately 14 cm in length.

**Figure 3:**
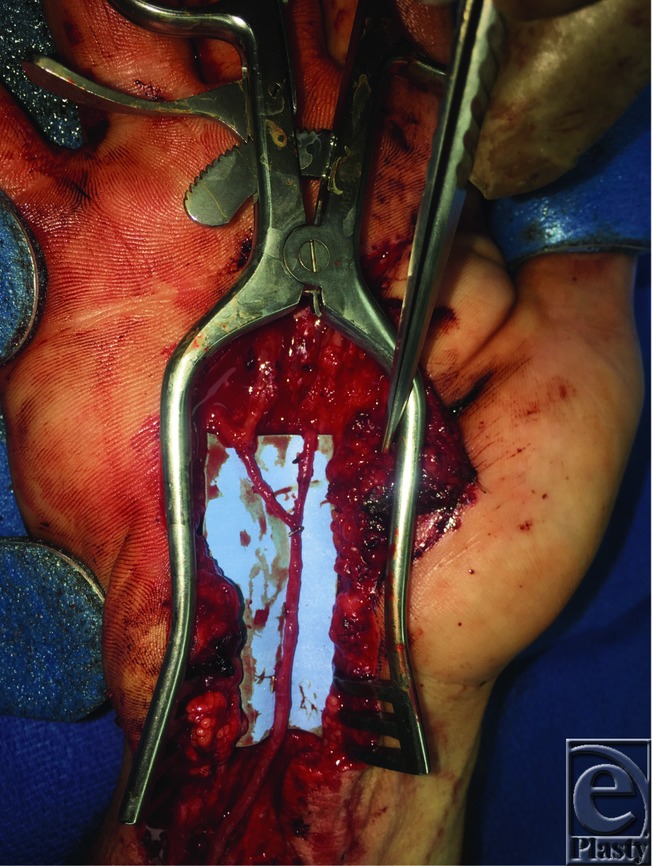
A close-up view of the reconstructed superficial palmar arch utilizing distal branches of the descending lateral circumflex femoral artery.

The arterial graft and recipient vessels were examined under the operative microscope. The proximal anastomosis was preformed first. The two branches at the distal end of the arterial graft were anastomosed to the superficial palmar arch stump, and common digital artery to the ring and small finger, respectively. A bolus of 5000 units of heparin was given intravenously before release of the microvascular clamps. Adequate pulsatile flow was achieved though the graft and confirmed with Doppler ultrasound to each finger (Figs 2 and 3, Video 1). The hand was then irrigated, and the ulnar nerve was explored to ensure that no injury had taken place. The skin was closed, and the patient's hand was placed into a dorsal splint.

**Figure 4:**
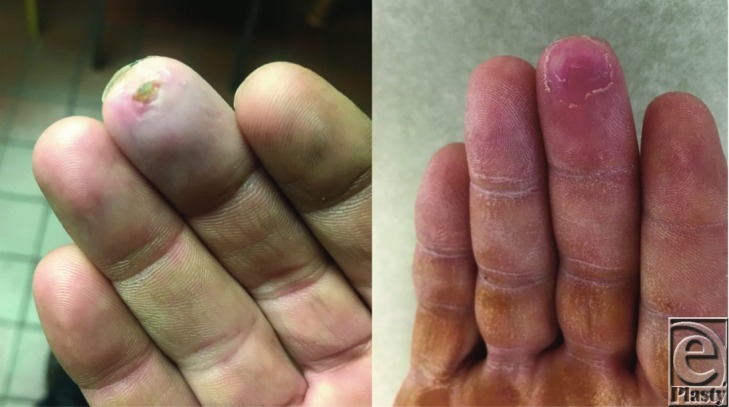
Preoperative image depicting ulceration of the tip of the right long finger (left). Postoperative image of the same site which has now healed, 1 month out from surgery following ulnar arterial reconstruction.

The patient remained in the hospital overnight for monitoring and was discharged the following morning on oral aspirin as the only anticoagulant. On follow-up examination 2 days later, strong Doppler signals were elicited at the ulnar artery, palmar arch, and radial and ulnar aspect of each digit. At 1 month, Doppler signals remained strong and the ulcer previously present on the right long fingertip had completely resolved (Fig 4, Video 2). During this visit he reported to be asymptomatic and has had complete resolution of the cold intolerance and burning pain of his right hand.

## QUESTIONS

1. What is hypothenar hammer syndrome?
2. Who gets hypothenar hammer syndrome?
3. How is hypothenar hammer syndrome typically treated?
4. What are the benefits of the lateral circumflex femoral artery as an interposition graft?

## DISCUSSION

Hypothenar hammer syndrome is a condition characterized by ulnar arterial insufficiency secondary to thrombosis or aneurysm.^1^ The suggested etiology of this relatively rare condition is repetitive blunt trauma to the hypothenar eminence, commonly in patients who use their hand as a “hammer.” Repetitive striking of the hypothenar eminence may lead to recurrent compression of the superficial palmar branch of the ulnar artery against the hamate bone, which is protected only by the palmaris brevis, aponeurosis, overlying subcutaneous fat, and skin.^2^ Anatomic variations of the deep and superficial palmar arch are common and may contribute to disease severity in HHS. In approximately 66% to 96% of patients, the superficial palmar arch is considered “complete,” supplying each of the fingers and the ulnar aspect of the thumb. Radial arterial contributions to the superficial palmar arch are less frequent and may exist in the form of direct communication with the distal end of the ulnar artery or a radial artery superficial palmar branch.^3^ These anatomic variations, as well as the extent of thrombosis or aneurysm, lead to a wide range of presenting symptoms seen in HHS.^1^ While the most common presentation is cold intolerance and pain, some patients may present with discoloration, ulceration, and necrosis of the affected digits, while others may be asymptomatic.

The overall incidence of HHS in the general population is unknown, although large prospective cohort studies have estimated it to be close to 1.6%.^2^^,^^4^ This condition has been widely associated with occupations such as mechanics, carpenters, roofers, and factory workers, with one study citing incidence rates as high as 14% in this “at-risk” population. This association with these occupations is thought to be due to the use of the hypothenar eminence as a hammer, or repetitive compression of this region with tools such as screwdrivers and wrenches.^4^ A recent systematic review identified 3 articles reporting significant positive relationships between repetitive occupational palmar trauma and the development of HHS. Additionally, HHS has been associated with frequent exposure to vibrating instruments, although evidence of this correlation in the literature is conflicting.^1^ Patients presenting with signs and symptoms of digital ischemia and an occupation predisposing them to repetitive palmar trauma should raise clinical suspicion for HHS.

Operative versus nonoperative management in HHS is often based on the acuity and severity of symptoms, as no established guidelines currently exist in the literature. Studies suggest that asymptomatic HHS may be treated conservatively with oral antiplatelet agents and vasodilators.^5^ Additional nonoperative management options include thrombolytics and chemical sympathectomy.^2^^,^^6^ A recent systematic review by Vartija et al^1^ evaluating 24 studies found that while partial symptom reduction was common following nonoperative management, complete resolution was identified in only 12% of patients. This finding led the authors to recommend initial nonoperative management with close follow-up and surgical intervention as needed.^1^ Several surgical options for HHS exist including sympathectomy, endovascular treatment, arterial excision without reconstruction, and reconstruction with vein or arterial grafts.^2^^,^^5^^,^^6^ In a 2011 study, Yuen et al^2^ proposed a surgical management algorithm based on timing of symptom onset and ischemia severity. In the setting of acute ischemia caused by segmental ulnar artery occlusion or aneurysmal thrombosis, endovascular fibrinolysis may be attempted. Ischemia onset beyond 2 weeks—or if thrombosis is unresolved following fibrinolysis—resection of the thrombosed segment may be indicated. Direct repair versus interposition graft may then be decided on the basis of the presence of patent outflow arteries and the remaining length of the ulnar artery following resection.^2^ Further investigation is necessary to establish consistent guidelines and effective treatment algorithms for the management of HHS.

Reconstruction of the ulnar artery in HHS may involve direct end-to-end arterial repair or an interpositional graft. Typically, a local venous graft harvested from the flexor aspect of the ipsilateral forearm has been the preferred option. In the HHS population—which typically consists of active young to middle aged males—durability and long-term patency is a primary concern.^7^ In the cardiovascular bypass literature, arterial autografts have been shown to be superior to venous grafts in terms of long-term patency. Additionally, these grafts maintain their physiologic responsiveness and secretion of endogenous vasodilators.^8^ The DLCFA is a reliable arterial graft option that has been described in the plastic surgery, cardiothoracic, and neurosurgical literature. The lateral circumflex femoral artery is derived from the profunda femoris and typically gives rise to an ascending, transverse, and descending branch. The DLCFA travels within the septum between the vastus lateralis and rectus femoris, forming the basis for the workhorse anterolateral flap (ALT).^9^ The modern plastic surgeon's extensive experience with the ALT flap and the dissection of the DLCFA makes this vessel a logical choice for an arterial graft. Additionally, donor site morbidity of the anterolateral thigh is often minimal. Harvest site complications may include thigh dysesthesia due to lateral femoral cutaneous nerve injury and scar dissatisfaction, both of which have been reported to be exceedingly rare.^10^ The few existing cases describing the use of arterial grafts in the setting of HHS have reported favorable outcomes, with patency and symptomatic improvement rates comparable or superior to that of venous grafts. In a 2015 small comparative study, de Niet and van Uchelen^7^ described 11 cases of HHS treated with DLCFA arterial graft. Each of the 11 grafts was found to be patent at 63 months’ follow-up, compared to only 9 of 32 venous grafts in the comparison group.^7^ While further large-scale comparative studies are necessary to elucidate the true risks and benefits of arterial grafts in the setting of HHS, preliminary studies show that this is a promising surgical option. The Supplemental Digital Content Video 1 shows lateral circumflex femoral arterial graft inset with strong pulsatile flow demonstrated throughout the extent of the graft, and the Supplemental Digital Content Video 2 shows postoperative Doppler signals of the ulnar artery, superficial palmar arch, and digital arteries at 1 month's follow-up.

## SUMMARY

Hypothenar hammer syndrome is a rare cause of ulnar arterial insufficiency that, if left untreated, may lead to severe digit ischemia and potential amputation. While end-to-end anastomosis and interposition venous grafts have been the mainstay of surgical management, arterial grafts utilizing the DLCFA may be a viable reconstructive option.
